# Characterization of Thermophilic Lignocellulolytic Microorganisms in Composting

**DOI:** 10.3389/fmicb.2021.697480

**Published:** 2021-08-11

**Authors:** María J. López, Macarena M. Jurado, Juan A. López-González, María J. Estrella-González, María R. Martínez-Gallardo, Ana Toribio, Francisca Suárez-Estrella

**Affiliations:** Unit of Microbiology, Department of Biology and Geology, CIAIMBITAL Research Center, ceiA3, University of Almería, Almeria, Spain

**Keywords:** culturome, xylanase, cellulase, ligninases, laccase, lignin peroxidase, thermotolerance

## Abstract

Composting involves the selection of a microbiota capable of resisting the high temperatures generated during the process and degrading the lignocellulose. A deep understanding of the thermophilic microbial community involved in such biotransformation is valuable to improve composting efficiency and to provide thermostable biomass-degrading enzymes for biorefinery. This study investigated the lignocellulose-degrading thermophilic microbial culturome at all the stages of plant waste composting, focusing on the dynamics, enzymes, and thermotolerance of each member of such a community. The results revealed that 58% of holocellulose (cellulose plus hemicellulose) and 7% of lignin were degraded at the end of composting. The whole fungal thermophilic population exhibited lignocellulose-degrading activity, whereas roughly 8–10% of thermophilic bacteria had this trait, although exclusively for hemicellulose degradation (xylan-degrading). Because of the prevalence of both groups, their enzymatic activity, and the wide spectrum of thermotolerance, they play a key role in the breakdown of hemicellulose during the entire process, whereas the degradation of cellulose and lignin is restricted to the activity of a few thermophilic fungi that persists at the end of the process. The xylanolytic bacterial isolates (159 strains) included mostly members of Firmicutes (96%) as well as a few representatives of Actinobacteria (2%) and Proteobacteria (2%). The most prevalent species were *Bacillus licheniformis* and *Aeribacillus pallidus*. Thermophilic fungi (27 strains) comprised only four species, namely *Thermomyces lanuginosus*, *Talaromyces thermophilus*, *Aspergillus fumigatus*, and *Gibellulopsis nigrescens*, of whom *A. fumigatus* and *T. lanuginosus* dominated. Several strains of the same species evolved distinctly at the stages of composting showing phenotypes with different thermotolerance and new enzyme expression, even not previously described for the species, as a response to the changing composting environment. Strains of *Bacillus thermoamylovorans*, *Geobacillus thermodenitrificans*, *T. lanuginosus*, and *A. fumigatus* exhibiting considerable enzyme activities were selected as potential candidates for the production of thermozymes. This study lays a foundation to further investigate the mechanisms of adaptation and acquisition of new traits among thermophilic lignocellulolytic microorganisms during composting as well as their potential utility in biotechnological processing.

## Introduction

Lignocellulosic materials, including agricultural and forestry waste, are defined as a valuable renewable carbon resource in the implementation of current biorefineries supported by Circular Economy. Its use is therefore considered as a useful and environmentally friendly alternative to traditional refineries based on the use of raw materials (RMs) derived from oil (Hemati et al., 2018). Some microorganisms have evolved different enzyme systems with specific catalytic strategies to degrade complex lignocellulose. In fact, lignocellulose depolymerization in natural ecosystems is highly efficient because of the synergistic action of multiple enzymes produced by taxonomically distinct microorganisms. For example, aerobic organisms, such as *Trichoderma* species and numerous actinomycetes, produce and secrete free lignocellulolytic enzymes, whereas anaerobic bacteria, such as *Clostridium thermocellum* and *Acetivibrio cellulolyticus*, integrate various cellulases (Cels) and xylanases (Xyls) into a large multienzyme complex (Bhalla et al., 2013).

One of the preferred environments for the recovery of lignocellulose-degrading microorganisms is the composting process (Jurado et al., 2014; Harindintwali et al., 2020). It has been extensively analyzed in order to determine the role of this specific group of microorganisms in the deconstruction of this type of recalcitrant polymer. Composting has been positioned for the last 25 years as a successful alternative for the treatment of organic waste, in which the biotransformation process is carried out by a complex microbial community (Schaub and Leonard, 1996; Insam and de Bertoldi, 2007; Moreno et al., 2021). During the process, heat is produced because of the energy generated by exergonic aerobic reactions derived from microbial metabolism. This leads the composting to evolve through different stages driven mainly by the temperature reached in the materials being transformed. The thermal phases that define a composting process are mesophilic, thermophilic, cooling, and maturation. Microbial activity is more intense during the first two stages (bio-oxidative phase), whereas the humification of the materials occurs mainly during the final phases of the process (maturation phase) (Villar et al., 2016). Temperature, therefore, will determine the structure and dynamics of microbial populations throughout composting, being also a key factor commonly used to confirm the process is running properly. Numerous data support that the temperature reached inside a composting pile favors the selection of an extremophile thermotolerant microbiota capable of intervening in the carbon and nitrogen recycling, acting on the lignocellulosic complex structure (Jurado et al., 2014; Hemati et al., 2018; Ince et al., 2020). Therefore, based on recent investigations, most microorganisms in composting could be defined as thermotolerant, as they become adapted to the changing temperature of the process (Moreno et al., 2021). These thermotolerant microorganisms are of special interest because of the thermostability of their enzymes (Bhalla et al., 2013), which makes them more competitive in industrial processes, compared to other more thermolabile microorganisms (Hemati et al., 2018).

During the last years, numerous authors have highlighted the biotechnological relevance of the populations of thermophilic organisms present in the composting process (Jurado et al., 2014; Ince et al., 2020). In fact, recent advances in molecular identification, metagenomics, and proteomics have made it possible to understand the metabolic diversity and functionality of different fungal and bacterial families involved in the process, in terms of their ability to degrade lignocellulosic fractions. In some cases, an approximation at the genus or even species level has been possible, showing a detailed profile of the microbiome of the composting piles, extremely dependent on the RMs and the operating conditions applied in each case (Langarica-Fuentes et al., 2014; Estrella-González et al., 2020; Ince et al., 2020). Other authors have described the capacities of microbial consortia from the “secretome” of lignocellulosic materials subjected to different thermal treatments, suggesting a metabolic coordination between different species with a view to deconstruction of the lignocellulose (Gavande et al., 2021). In any case, most of the studies described, although novel because they are based on the use of genomic mining and powerful data platforms, do not allow knowing the thermal tolerance range of the identified taxa, nor the actual capacity to deconstruct lignocellulosic fractions.

Therefore, the objectives of this work were: (i) to quantify thermophilic microorganisms and their lignocellulolytic representatives during a plant waste-based composting process, in parallel to the analysis of the evolution of the degradation of the lignocellulose; (ii) to analyze the ability to degrade cellulose (CEL), hemicellulose (HC), and lignin (LIG), the range of thermal tolerance, and identity by sequencing of the lignocellulolytic thermophilic bacteria and fungi from the different stages of the process; and (iii) to select specific strains with potential application for the production of thermostable enzymes.

## Materials and Methods

### Composting and Sampling Strategy

The composting parameters and procedure were performed according to López-González et al. (2013). A composting pile of 3.0-m length × 1.5-m width × 1.0-m height was prepared by mixing shredded (<3 cm) tomato plant waste (stalks and leaves) with pine chips (50:50 wt/wt) in order to get a carbon-to-nitrogen ratio around 25. The pile was subjected to forced aeration (7.5–9.0 L/kg every 4 h) to maintain oxygen concentration inside the pile over 10%. The air was supplied from the bottom of the pile through perforated PVC tubes connected to a Lowarda CEAM-7013 pump (Montecchio Maggiore, Italy). Turnings were performed using a tractor shovel when the temperature inside the pile dropped for three consecutive days (approximately every week). The moisture content was initially set between 50 and 55% (wt/wt), and it was maintained within this range by watering during turning operations. These management operations were applied during the bio-oxidative phase (2 months) until the material cooled down. After this period, the piles were statically maintained in maturation for an additional period (4 months), so the process lasted for a total of 6 months. Temperature values inside the piles were continuously measured using a 50-cm-long Pt 100 temperature probe model MPT2 (Lexitron-Guemisa, Madrid, Spain) connected to a data logger.

Samplings were carried out at different stages of the composting process, according to the thermal values prevailing inside the piles, including RMs, mesophilic stage (MES), thermophilic stage (THER), cooling stage (COOL), maturation stage (MAT), and final product (Table 1). Composite samples (500 g) were collected at each sampling time by properly mixing and homogenizing subsamples extracted from nine different locations inside each pile. Each sample was divided into two equal parts; one of them was kept at 4°C for further chemical analysis, whereas the second portion was immediately used for the microbiological analyses.

**TABLE 1:** Sampling times and main characteristics of material.

Composting stage*	**Day**	Temperature** (°C)	**Organic matter (%)**	C/N***	**pH**
RM	0	24.1 ± 2.3	65.9 ± 3.5	27.7 ± 2.4	7.9 ± 0.1
MES	5	41.8 ± 5.1	62.1 ± 2.1	24.3 ± 1.0	8.7 ± 0.1
THER	16	59.4 ± 6.0	61.5 ± 2.6	24.3 ± 0.9	8.5 ± 0.2
COOL	63	36.1 ± 3.4	60.7 ± 2.0	21.1 ± 1.1	8.6 ± 0.1
MAT	168	27.4 ± 1.4	50.9 ± 4.1	15.9 ± 2.1	8.6 ± 0.1
FP	189	20.6 ± 1.0	43.9 ± 2.2	13.3 ± 0.7	8.5 ± 0.1

*^∗^Composting stage: RM, raw material; MES, mesophilic; THER, thermophilic; COOL, cooling; FP, final composting product.*

*^∗∗^Temperature values are the mean and SD for the period between the corresponding stage and the previous one (except for RM in which average values of the same material are included n = 3).*

*^∗∗∗^C/N, carbon-to-nitrogen ratio reached at each stage.*

### Characterization of Solid Compost Samples: Lignocellulosic Fractions

Cellulose, HC, and LIG fractions were determined using a fiber analyzer ANKOM200/220 (Ankom Technology, United States) according to the methods established by Ankom Technology for Acid Detergent Fiber, Neutral Detergent Fiber, and Acid Detergent Lignin.^1^ Holocellulose was expressed as the sum of CEL and HC values. Losses of lignocellulosic fractions (HC, CEL, and LIG) were calculated from the initial (A1) and final (A2) ash contents according to the equation of Paredes et al. (2000), where P1 and P2 were the initial and final concentrations of lignocellulose fraction (mg/g).

%Loss=100-100×((A1×P2)/(A2×P1))

### Analysis of Thermophilic Microorganisms

#### Counts, Isolation, and Maintenance of Microorganisms

Suitable culture media and incubation temperatures were employed for the isolation and enumeration of thermophilic strains. Besides bacteria and fungi, the specific bacterial group of actinobacteria was analyzed separately because of its important role in the degradation of lignocellulose during composting (Steger et al., 2007; Jurado et al., 2014). Thermophilic actinobacteria were cultured in Actinomycete Isolation Agar Glycerol (Difco, Becton, Dickinson & Co., MD, United States) and incubated at 55°C for 4–5 days. Thermophilic bacteria were cultured in standard Nutrient Agar (NA; Cultimed, Spain) at 55°C for 2–3 days. Thermophilic fungi were cultured in Rose Bengal Chloramphenicol Agar plates (Cultimed, Spain) at 55°C for 4–5 days. Fresh samples (10 g) were suspended in 90 mL sterile saline solution (0.9% NaCl in distilled water) and shaken [150 revolutions/min (rpm)] at room temperature for 30 min. Then, 10-fold serial dilutions in sterile saline solution were performed, and 100 μL from dilutions was spread out over Petri plates with the required culture media. Counts were expressed as colony-forming units per gram of sample dry weight [colony-forming units (CFU)/g]. Each different colonial type (according to size, morphology, pigmentation, and texture) in each plate was separately counted and then transferred to a new plate with fresh medium. Plates were incubated (same times and temperatures as before indicated), checked for purity, and stored at 4°C (working pure cultures) or preserved in cryoballs Cryoinstant (Deltalab, Spain) for long-term conservation. Pure cultures were photographed, and all morphotypes were compared. In addition, microscopic observations, Gram and spore stains, and catalase and oxidase tests (Gerhardt et al., 1994) were performed in order to eliminate repeated isolates.

#### Microbial Enzymatic Profile: Cellulolytic, Xylanolytic, and Ligninolytic Activity

The isolated microorganisms were tested for the expression of ligninolytic (Lig), cellulolytic (Cel), and xylanolytic (Xyl) activities. All the assays were performed on plates with solid medium containing the appropriate substrate for the specific activity as described below. For bacteria and actinobacteria, the plates were inoculated with 25 μL droplets of biomass suspension made in 500 μL sterile saline solution (NaCl 0.9%) from microorganism grown on one APHA (Cultimed, Spain) agar slant for 48 to 72 h. In the case of fungi, pieces of 6-mm diameter from a 96-h culture on PDA (Potato Dextrose Agar, Scharlab, Spain) were placed in the plates. The inoculated media were incubated at 50°C for 5 days (Xyl activity) and 7–10 days (Cel and Lig enzyme activities) and checked for the presence of the activity. Cel and Lig microorganisms showed decolorization around the colony grown on 0.5% CEL plus 0.005% aniline blue black (Kauri and Kushner, 1988) and Poly R-478-containing (Freitag and Morrell, 1992) agar plates, respectively. A clear halo around colonies demonstrated Xyl activity on 0.5% (wt/vol) xylan-containing medium.

#### Thermotolerance Profiles of Isolates

The range of growth temperatures and optimal growth temperature of strains within the thermal interval of 20, 30, 40, 50, and 60°C was determined according to Moreno et al. (2021). Plates of NA (Scharlab, Spain) (bacteria) or PDA (fungi) were inoculated by streaking sterile swabs previously soaked in fresh liquid cultures on NB (bacteria) or PDB (fungi). After incubation at the different temperatures for 48–72 h (bacteria), 96 h (fungi), and 96–120 h (actinobacteria), tests were considered positive when visible microbial growth was evident, so a range of growth temperature was determined. To determine optimal microbial growth at the selected temperatures, a microtiter assay based on Resazurin reduction was employed as described by Moreno et al. (2021) based on the procedure proposed by Chadha and Kale (2015) and Vega et al. (2012). Three replicates were used for each combination strain/temperature.

#### Production and Quantification of Xyl, Cel, and Ligninase Enzymatic Activities

Xylanase and Cel production were induced by culturing selected microorganisms in 100-mL flasks with 20 mL minimal salt medium (Janshekar et al., 1982), added to 0.5% (wt/vol) beechwood xylan (Apollo Scientific) or carboxymethylcellulose (CMC) (Sigma-Aldrich) as the sole carbon and energy source, respectively. For the production of ligninases, i.e., laccase (Lac), LIG peroxidase (LiP), and manganese peroxidase (MnP), 20 mL of Kirk medium (Kirk et al., 1986) that contained veratryl alcohol as inducer, was used. The production media were inoculated with 1-cm^2^ plug of a 7-day-old fungal mycelium precultured in PDA (Potato Dextrose Agar, Scharlab, Spain) or 2% (vol/vol) bacterial suspensions in 0.9% NaCl with OD_600_ 0.5 from a preculture incubated at 50°C in NA (Scharlab, Spain). Cultures were incubated at 50°C statically (fungi) or shaking at 110 rpm (bacteria), for 7 days. After the incubation time, 10 mL was centrifuged at 10,000 *g* for 10 min to obtain the supernatant with the crude extract in which the enzymatic activities were analyzed.

Xylanolytic and Cel activities were determined by measuring the reducing sugars released using the 3,5-dinitrosalicylic acid (DNS) method, with xylose and glucose as standard, respectively (Miller, 1959). Xyl reaction mixture contained 250 μL of the crude enzymatic extract and 250 μL of substrate consisting of 1% (wt/vol) beechwood xylan (Sigma) in phosphate–citrate buffer (50 mM at pH 6.5) (He et al., 1993). Cel reaction mix consisted of 250 μL crude enzymatic extract and 250 μL of substrate containing 1% (wt/vol) sodium CMC (Sigma) in sodium acetate buffer (50 mM at pH 5). Controls were prepared with enzyme added after DNS reagent addition (1.5 mL). After incubation at 50°C for 1 h, the released reducing sugars were assayed by DNS method spectrophotometrically estimated (Shimadzu UV-160A) at 550 nm. One unit of Xyl and Cel activities (U) was defined as the amount of enzyme required to release 1 μmol of xylose and glucose, respectively, per minute under assay conditions.

Laccase, LiP, and MnP were analyzed in reactions with a final volume of 2 mL. Lac was determined by monitoring the oxidation of syringaldazine at 525nm for 2 min (ε525 = 65,000 M^–1^ cm^–1^) (Orth et al., 1993). The reaction mixture comprised 200 μL syringaldazine (0.216 mM), 500 μL citrate–phosphate buffer (400 mM, pH 5.2), and 500 μL enzymatic extract. LiP was performed by measuring the oxidation of veratryl alcohol to veratraldehyde at 310 nm for 2 min (ε310 = 9,300M^–1^ cm^–1^) (Orth et al., 1993). The reaction mixture contained 200 μL veratryl alcohol (20 mM), 200 μL sodium tartrate buffer (250 mM, pH 2.5), 200 μL hydrogen peroxide solution (4 mM), and 500 μL enzymatic extract. MnP activity was performed by measuring the oxidation of Mn^2+^ to Mn^3+^ at 238 nm (ε238 = 6,500 M^–1^ cm^–1^) (Camarero et al., 1999). The reaction volume contained 200 μL manganese sulfate (1 mM), 200 μL sodium tartrate buffer (1 M, pH 5), 200 μL hydrogen peroxide solution (1 mM), and 500 μL enzymatic extract. All enzymes assays were carried out using a UV array spectrophotometer (Shimadzu UV-160A). One unit of enzyme activity was defined as the enzyme required for the formation of 1 μM of the product per minute under the conditions of the reaction.

### Identification of Microorganisms

The identification protocol was based on partial or nearly full-length 16S rRNA gene (bacteria, including actinobacteria) and 5.8S-ITS region (yeast and fungi) sequence analysis. The 16S rRNA genes of bacteria were amplified using universal primers: 27F (5′-AGAGTTTGATCATGGCTCAG-3′) and 1492R (5′-GGTTACCTTGTTACGACTT-3′) (Weisburg et al., 1991). The 5.8S-ITS region of fungi and yeast was amplified using primers ITS1 (5′-TCCGTAGGTGAACCTGCGG-3′) and ITS4 (5′-TCCTCCGCTTATTGATATGC-3′) (White et al., 1990). Amplified PCR products were checked by gel electrophoresis on 1% agarose gel and purified using the Diffinity Rapid Tips (Sigma--Aldrich). The clean DNA was sequenced by capillary sequencer ABI Hitachi 3500 Genetic Analyzer (Applied Biosystems). The forward and reverse sequences were edited, assembled, and aligned using Sequence Scanner v1.0 (Applied Biosystem), Reverse Complement,^2^ Clustal Xv2.0.11 (Larkin et al., 2007), and MEGA 5 v5.2 (Tamura et al., 2011). The sequences were then compared for similar nucleotide sequences with the BLAST search of the National Center of Biotechnology Information (NCBI).^3^

### Data Analysis

Three independent replicates were used in all analyses, and the data obtained were subjected to statistical analysis using Statgraphics Centurion XVIII (StatPoint, Inc., Virginia). Welch analysis-of variance and Games–Howell multiple comparisons were applied to compare mean values of the optimal growth temperature of the microorganisms for the different levels of composting stage or taxon.

## Results and Discussion

### Abundance of Lignocellulose-Degrading Thermophiles During Composting and Degradation of Lignocellulosic Fractions

**FIGURE 1:**
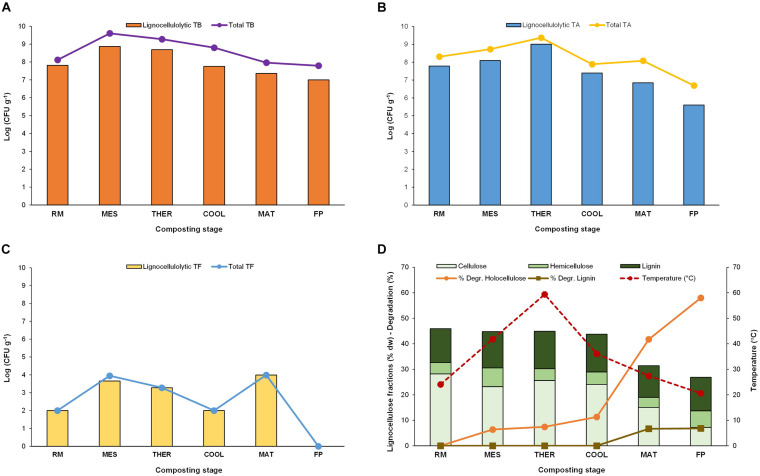
Counts of total and lignocellulolytic thermophilic **(A)** bacteria (TB), **(B)** actinobacteria (TA), and **(C)** fungi (TF). **(D)** Thermal values and lignocellulose fraction content (%) and degradation (%). Composting stage: RM, raw material; MES, mesophilic; THER, thermophilic; COOL, cooling; FP, final composting product.

Thermophilic bacteria, actinobacteria, and fungi at each composting stage, as well as the levels of lignocellulolytic representatives for each group, are shown in Figure 1 (Supplementary Table 1). Total thermophilic bacteria and actinobacteria peaked at MES and THER and maintained high values, in the range of 10^6^–10^8^ CFU/g, throughout the process. In contrast, thermophilic fungi were far less abundant, with levels not exceeding a maximum of 10^3^ CFU/g at MES and THER, and were absent in the final compost likely because of competition with mesophilic microbial population (López-González et al., 2015; Moreno et al., 2021). Noteworthy was the fact they experienced a final increase at MAT, after decreasing at COOL, which is a trend also reported earlier in composting of anaerobic digestates (Di Piazza et al., 2020). The thermophilic lignocellulolytic bacteria and actinobacteria count was approximately 10 log units lower than total thermophilic but showed the same trend at the composting stages (Figures 1A, B). In the case of fungi, the lignocellulolytic population was nearly 100% of the total thermophilic fungi counts (Figure 1C). Although thermophilic microorganisms are not uniquely responsible for the biodegradation of lignocellulose during composting, they play a key role because they actively grow at the THER when it has been reported that lignocellulose degrades at a faster rate (Xiao et al., 2009; Qiao et al., 2019). In the present study, the degradation of holocellulose (sum of CEL and HC) took place mainly after COOL, reaching a net value of 58% at the end (Figure 1D) (Supplementary Table 1). It started from the early composting, at MES (6%), and slightly increased right after THER. Considering that MES and THER lasted for 5 and 11 days, respectively, and the time between cooling and the final composting product was 126 days (Table 1), the faster holocellulose degradation occurred at MES, when the daily degradation rate was 1.5, in contrast with the later period in which the value was 0.5. Notwithstanding this, it is at THER when the thermophilic microorganisms are the unique active organisms and the sole ones responsible for the degradation of the lignocellulose that could lead to loosening its structure facilitating further degradation. Noticeably, the degradation of LIG was negligible up to MAT in which 7% of this polymer was degraded, an activity that matched with the late increase of thermophilic fungi counts.

### Lignocellulolytic Thermophilic Bacteria and Fungi

The thermophilic isolates having lignocellulose-degrading activity consisted of 186 strains, including 159 bacteria and 27 fungi, for whom the specific composting stage when they were obtained was known. Bacterial strains only had Xyl activity, whereas among the fungi, Cel and Lig activities were also found for some strains. All isolates were identified by sequencing 16S rRNA or 5.8S-ITS region for bacteria and fungi, respectively. In addition, the range of growth at temperatures between 20 and 60°C as well as the optimal temperature for growth was determined for each isolate. Publicly available datasets analyzed in this study can be found in Mendeley Data, V1 (doi.org/10.17632/265tjt9f4m.1).

**FIGURE 2:**
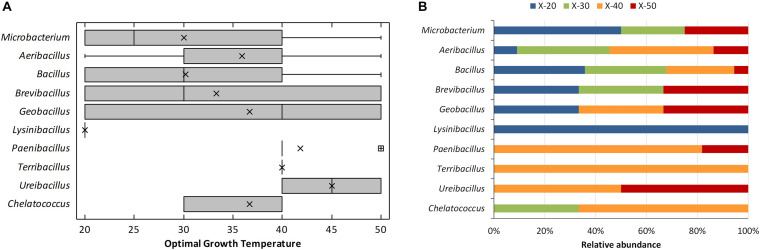
Optimal growth temperatures of the thermophilic xylanolytic bacterial genus isolated from compost. **(A)** Box-and-whisker plot summarizing the range of optimal growth temperature for the different strains of each genus; mean values (x), median (I), and outliers (□) are represented; **(B)** relative abundance of xylanolytic strains for each genus having the optimal growth temperature: 20°C (X-20), 30°C (X-30), 40°C (X-40), and 50°C (X-50).

The Xyl bacterial isolates included mostly members of the phylum Firmicutes (96%), besides a few representatives of the phyla Actinobacteria (2%) and Proteobacteria (2%) (Supplementary Table 2). Actinobacteria strains were identified as *Microbacterium hydrocarbonoxydans* and *Microbacterium sediminis*, whereas the Proteobacteria were all *Chelatococcus daeguensis*. Within the Firmicutes, there were representatives of 24 species belonging to seven genera, all of the Class Firmicutes, with a clear dominium of *Bacillus* (109 strains) followed by *Aeribacillus* (22 strains), *Paenibacillus* (11 strains), *Brevibacillus* (3 strains), *Geobacillus* (3 strains), *Ureibacillus* (2 strains), *Lysinibacillus* (1 strain), and *Terribacillus* (1 strain). Among the 27 different species detected, most isolates (70%) belonged to five of them: *Bacillus licheniformis* (49 strains), *Aeribacillus pallidus* (22 strains), *Bacillus thermoamylovorans* (17 strains), *Bacillus pumilus* (12 strains), and *Paenibacillus ginsengihumi* (10 strains). Approximately 87% of the bacterial strains grew at thermotolerant temperature ranges (20–60°C), whereas 4% could be considered true thermophiles, i.e., those unable to grow at temperatures below 40°C. These included members of the species *A. pallidus* and *B. thermoamylovorans*. The dominium of thermotolerant microorganisms during composting has been already demonstrated for the total microbial population (Moreno et al., 2021) and also applies to thermophilic Xyl bacteria. The variations in optimal growth temperature obtained for the different strains of each genus are shown in Figure 2A. Approximately 50% of the isolates grew optimally at 40°C or 50°C (Figure 2B). The largest variability for this trait was obtained for strains of the genera from the phylum Firmicutes, *Brevibacillus*, *Geobacillus*, *Bacillus*, *Aeribacillus*, and the Actinobacteria *Microbacterium*, which had representatives in both extremes of the temperatures tested. All these Firmicutes genera have been earlier reported to grow in a wide range of temperatures (Bhattacharya and Pletschke, 2014; Verma et al., 2019), but *Microbacterium* usually grows at temperature lower than 44°C (Fidalgo et al., 2016). *Chelatococcus* strains were optimal at 30°C and 40°C, whereas it was over 40°C for the strains of the genera *Paenibacillus*, *Terribacillus*, and *Ureibacillus*. The unique representative of *Lysinibacillus* had optimal growth at 20°C (Figure 2A), that is, in the range of growth temperature of the genus (16°C–45°C) (Ahmed et al., 2007). Xyl activity, thermotolerance, and thermophilia are common traits among Firmicutes, especially for bacilli and related genera (Bhattacharya and Pletschke, 2014). In fact, *Geobacillus*, *Paenibacillus*, and *Bacillus*, our predominant isolates, are the most widely reported thermophilic bacteria for their ability to produce thermostable Xyl (Verma et al., 2019). Noteworthy, the second most abundant bacilli isolate, *A. pallidus* (formerly *Geobacillus pallidus*), is thermophilic and alkalitolerant (Minana-Galbis et al., 2010) for whom no Xyl activity has been reported in prior studies. That also applies to the Alphaproteobacteria *C. daeguensis*, a typical mesophilic bacteria (Yoon et al., 2008), for which thermophilic strains have been recently reported (Wei et al., 2017) while having no Xyl activity detected to date. Actinobacteria also harbor Xyl enzymes; although *Microbacterium* is not the typical genus of that phylum for such activity, some strains having Xyl activity have been isolated from cricket gut (Kim et al., 2014). No Cel and Lig activity was found among the bacterial isolates, but this requires a deeper screening. Thermophilic bacteria belonging to *Bacillus* and *Geobacillus* are known to produce thermostable Cels (Patel et al., 2019) and the LIG-degrading enzyme Lac (Bugg et al., 2011; Chauhan et al., 2017).

**FIGURE 3:**
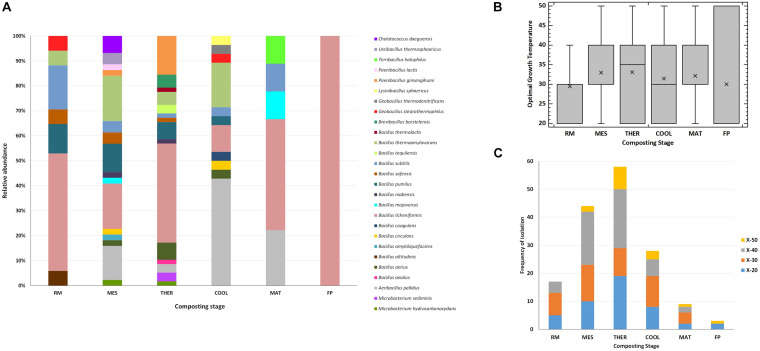
Thermophilic xylanolytic bacterial strains at the different stages of composting. **(A)** Relative abundance of the species; **(B)** Box-and-whisker plot with the range of optimal growth temperature for the different strains, mean values (x), median (I) and outliers (□) are represented; **(C)** frequency of isolation of the bacterial phenotypes: xylanolytic strains with optimal growth at 20°C (X-20), 30°C (X-30), 40°C (X-40) and 50°C (X-50). Composting stage: RM, raw material; MES, mesophilic; THER, thermophilic; COOL, cooling; FP, final composting product.

Considering the different stages of composting, the widest number of thermophilic bacterial Xyl strains and the largest diversity of species were obtained from mesophilic (44 strains of 16 species) and thermophilic (58 strains of 15 species) stages, whereas at maturation and final composting product, only nine strains of five species and three strains of one species, respectively, showed Xyl activities (Figure 3A). The unique Proteobacteria, *C. daeguensis*, was recovered from the MES. The two species of Actinobacteria, *M. hydrocarbonoxydans* and *M. sediminis*, were isolated from MES and THER, whereas the species of the phylum Firmicutes dominated over all stages. *B. licheniformis* strains accounted for 50% of isolates, except at MES and COOL. At the MES, *A. pallidus*, *B. licheniformis*, and *B. thermoamylovorans* together represented 50%, whereas at the COOL, *A. pallidus* dominated. In the final composting product, only *B. licheniformis* was isolated. Furthermore, the optimal mean growth temperature of the strains, as well as the range of phenotypes for such traits, showed a tendency to increase throughout the process (Figure 3B). This clearly relates to the adaptation of the microbial community to the thermal variations during composting (Moreno et al., 2021). The strains isolated from the RMs had an optimal growth average of 30°C with the maximum at 40°C. From the MES, the average temperature increased and the optimal growth temperature among the strains covered the whole range tested. The phenotypes with optimal growth at 50°C appeared at the MES and, as expected, increased at thermophilic to decrease again at cooling and maturation, but even at these cold stages, they were present (Figure 3C). The phenotypes having optimal growth temperature at or greater than 40°C represented 50% of the isolates at MES and THER and approximately 30% in the other stages. Thus, Firmicutes account for the most prevalent group of Xyl thermophiles during composting, being represented by diverse members of the genus *Bacillus*. They also have a wide spectrum of thermostability, which allows them to survive and remain metabolically active over all stages. Consequently, this group should play a crucial role in the breakdown of HC during the entire process. Similar conclusions have been obtained using metagenomics analysis (Antunes et al., 2016; Gavande et al., 2021). Furthermore, it was noticeable that many bacterial isolates having the same accession number showed different thermal phenotypes at the stages of composting or even at the same stage (Table 2). Although with some exceptions, most strains with typical thermophilic phenotypes, i.e., those unable to grow below 30°C or 40°C and optimum at 40°C or 50°C, were recovered from THER and, mainly, COOL, whereas those capable to grow over the full range of temperatures tested (20°C–50°C) were found nearly in all the stages. This may reflect the phenotypic plasticity triggered by the changing composting environment (Moreno et al., 2021).

**TABLE 2:**
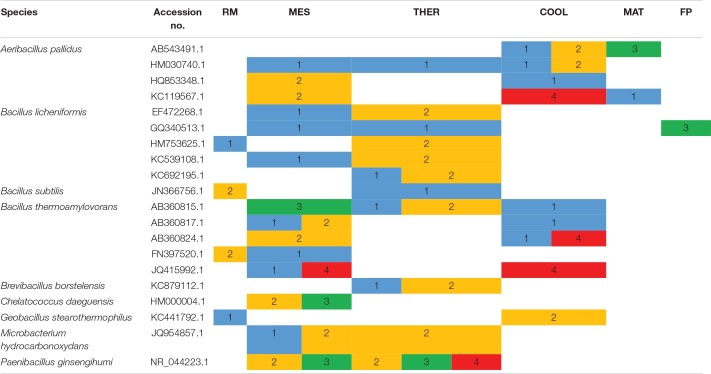
Thermal phenotypes of thermophilic xylanolytic bacterial strains with the same accession number detected at the different composting stages: RM, raw material; MES, mesophilic; THER, thermophilic; COOL, cooling; FP, final composting product.

*Thermal phenotypes: (1) range (20°C–50°C/20–60°C), optimum (20°C, 30°C); (2) range (20°C–60°C), optimum (40°C, 50°C); (3) range (30°C–60°C), optimum (40°C, 50°C); (4) range (40°C–60°C/50°C–60°C), optimum (40°C, 50°C).*

**FIGURE 4:**
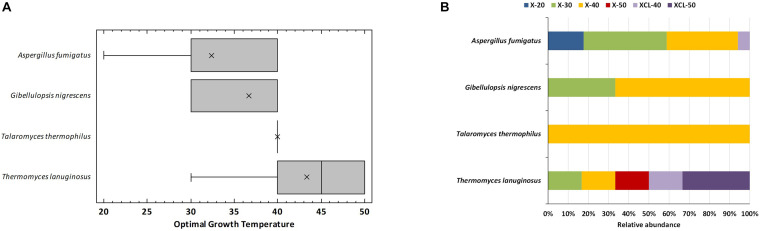
Optimal growth temperatures of the thermophilic fungi isolated from compost. **(A)** Box-and-whisker plot summarizing the range of optimal growth temperature for the different strains of each species; mean values (x), median (I), and outliers (□) are represented; **(B)** relative abundance of strains for each species having the optimal growth temperature and enzyme activity: xylanolytic strains with optimal growth at 20°C (X-20), 30°C (X-30), 40°C (X-40), and 50°C (X-50), and xylanolytic-cellulolytic-ligninolytic strains with optimal growth at 40°C (XCL-40) and 50°C (XCL-50).

The sequencing of the 27 fungal strains resulted in four known species of the phylum Ascomycota: *Thermomyces lanuginosus* (6 strains), *Talaromyces thermophilus* (renamed as *Thermomyces dupontii*) (1 strain), *Aspergillus fumigatus* (17 strains), and *Gibellulopsis nigrescens* (3 strains) (Supplementary Table 3). All fungal isolates were Xyl, whereas three *T. lanuginosus* strains and one *A. fumigatus* exhibited also Cel and Lig activities, which are not common for *T. lanuginosus*. The four fungal species detected have been earlier reported in compost using culture-dependent or independent techniques (López-González et al., 2015; Tiquia-Arashiro, 2019), and all except *G. nigrescens* are well-known as the most frequent thermophilic fungal taxon recovered from such an environment (Hutchinson et al., 2019). *G. nigrescens* has been earlier defined as a typical mesophilic (Zare et al., 2007) for whom no Xyl activity has been reported to date. Most strains grew at temperature ranges of thermophilic fungi (20°C–60°C), two *A. fumigatus* strains were mesophilic (20–30°C), and one strain of the same species was a true thermophilic growing only at a temperature greater than 40°C. Independent-samples Welch *t* tests revealed no significant differences between species for optimal growth temperature because of the wide range of values exhibited among isolates. However, it was clear that *T. lanuginosus* strains had higher optimal growth temperature (average value of 43°C) than the other species, whose optimal growth temperature never surpassed 40°C (Figure 4A). Thermophilic fungi are the ones whose growth temperature ranges from 20 to 62°C with the optimum at 35–55°C, whereas thermotolerant are the ones with a growth temperature range of 20–55°C (Maheshwari et al., 2000; Hutchinson et al., 2019). Excluding the *A. fumigatus* mesophilic isolates, 48% of the isolates were thermophilic with representatives of all species and 44% thermotolerant. The different isolates were categorized into six phenotypes according to the optimal temperature for growth and the lignocellulolytic activity, as follows: Xyl strains with optimal growth at 20, 30, 40, or 50°C (X-20, X-30, X-40, X-50), and those having also Cel and Lig activities with optimal growth at 40 or 50°C (XCL-40 and XCL-50). The distribution of these phenotypes among the four species is shown in Figure 4B. The widest range of phenotypes was obtained for *T. lanuginosus*, which had representatives of all phenotypes, except X-20. Noteworthy, strains of this species were unique, having optimal growth at 50°C and X (Xyl activity) or XCL (Xyl, Cel, and Lig activities). Both *A. fumigatus* and *T. lanuginosus* have a degradation system essential for adaptation during rising temperatures or nutrient limitation in composting materials (Mchunu et al., 2013; Kwon-Chung and Sugui, 2013), which explains their ubiquitous distribution and wide range of phenotypes.

**FIGURE 5:**
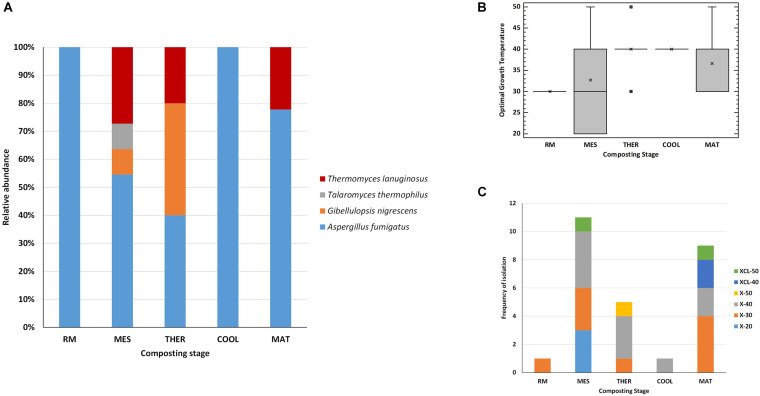
Thermophilic lignocellulolytic fungal strains at the different stages of composting. **(A)** Relative abundance of the species; **(B)** box-and-whisker plot summarizing range of optimal growth temperature for the different fungal strains; mean values (x), median (I), and outliers (□) are represented; **(C)** frequency of isolation of the phenotypes: xylanolytic strains with optimal growth at 20°C (X-20), 30°C (X-30), 40°C (X-40), and 50°C (X-50), and xylanolytic-cellulolytic-ligninolytic strains with optimal growth at 40°C (XCL-40) and 50°C (XCL-50). Composting stage: RM, raw material; MES, mesophilic; THER, thermophilic; COOL, cooling; FP, final composting product.

In terms of occurrence of the fungal species at the different composting stages (Figure 5A), *A. fumigatus* was the most frequently recovered taxon from all stages up to maturation, and it was the unique thermophilic lignocellulolytic fungi in the RM and COOL. The dominance of this species relates to its capability to survive and propagate successfully under a wide range of environmental conditions (Kwon-Chung and Sugui, 2013). It has been also described as the pioneer thermophilic mycobiota of mushroom compost (Hutchinson et al., 2019). Moreover, it has a strong ability to degrade lignocellulose (Liu et al., 2013; de Gouvêa et al., 2018). The secretome of *A. fumigatus* has been characterized as having Cel and Xyl activities, among others, but no ligninases (Liu et al., 2013; Vivek-Ananth et al., 2018; Wang et al., 2018). *T. lanuginosus* isolates were obtained at the MES and THER and appeared again at the MAT. It has been demonstrated by integrated meta-omics that this fungus can secrete a large amount of Xyl during composting and dominated the fungal population in maize straw compost (Zhang et al., 2015). *G. nigrescens* was found in MES and THER, whereas *T. thermophilus* was exclusively isolated from the MES. Accordingly, the MES was the only one in which representatives of all four species were present. No thermophilic lignocellulolytic fungus was isolated from the final compost. Moreover, as the composting proceeds, the average optimal growth temperature of the strains increased from 30 to 40°C, and the range of values was very variable for the strains isolated from MES and MAT (Figure 5B). Similarly, the phenotypes were more diverse at MES and MAT (Figure 5C). It was noticeable that XCL phenotypes, i.e., strains that have Xyl, Cel, and Lig activity, were mainly recovered at the MAT, at which degradation of LIG (7%) was evidenced (Figure 1D). Antunes et al. (2016), by analyzing the transcriptional profile of genes predicted to be involved in lignocellulose degradation, also demonstrated that Lig activity reaches a peak only at the end of the composting, although no fungal ligninases (Lacs, MnP, and LiP) were detected. These results indicate that lignocellulosic biomass deconstruction occurs synergistically and sequentially, with HC being degraded preferentially to CEL and LIG, as also seen in our study. It is also noteworthy to mention that, as occurred for the bacteria, some strains with the same accession number had different phenotypes even when isolated from the same sampling (Table 3). These fungi activate pathways that facilitate adaptation to the composting environment, and they may be able to adapt to several factors including nutrients, gases, water activity, and competing species, besides just high temperature (Maheshwari et al., 2000; Kwon-Chung and Sugui, 2013; Wang et al., 2018), and as the composting proceeds, the strains become more selective. Overall, the results revealed that the thermophilic lignocellulolytic fungal culturome was primarily dominated by *A. fumigatus* and *T. lanuginosus*.

**TABLE 3:**
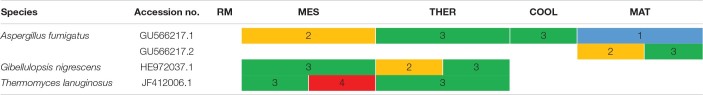
Thermal phenotypes of thermophilic lignocellulolytic fungal strains with same accession number detected at the different composting stages: RM, raw material; MES, mesophilic; THER, thermophilic; COOL, cooling; FP, final composting product.

*Thermal phenotypes: (1) range (20°C–50°C/20°C–60°C), optimum (20°C, 30°C); (2) range (20°C–60°C), optimum (40°C, 50°C); (3) range (30°C–60°C), optimum (40°C, 50°C); (4) range (40°C–60°C/50°C–60°C), optimum (40°C, 50°C).*

### Production of Enzymes

In order to determine the biotechnological potential for lignocellulolytic enzyme production by isolates analyzed in the exploratory analysis previously described, thermophilic bacterial and fungal strains were cultured for 7 days in liquid media containing commercial inducers as carbon source (i.e., CMC, xylan), and the enzymatic activity was measured after reaction of the crude enzymatic extract with the corresponding substrate at 50°C. The bacteria *B. thermoamylovorans* 1141 and *G. thermodenitrificans* 3781 and the fungi *T. lanuginosus* 5718 and *A. fumigatus* 5731 were selected for this analysis. This selection was based on previous knowledge on enzyme production or lack of it in literature (Koeck et al., 2014; Winger et al., 2014; de Gouvêa et al., 2018; Verma et al., 2020), the persistence during composting, the set of enzymes detected, and the range and optimal growth temperature for the particular strain. For bacteria, only Xyl production was determined, whereas for the fungi, Cel (CMCase) and ligninases (Lac, LiP, and MnP) were also analyzed along with, as shown in Table 4. All the strains selected were able to grow at a wide range of temperatures, had optimal growth at or greater than 40°C, and their enzymes were active at 50°C. These are valuable properties when considering the potential applications of the microorganisms and their enzymes (Bhalla et al., 2013; Bala and Singh, 2019; Patel et al., 2019).

**TABLE 4:** Production of enzymes by selected thermophilic isolates.

**Strain**	**Growth temperature (°C)**		**Enzymatic activity (U/mL)**				
	**Range**	**Optimal**	**Cellulase**	**Xylanase**	**Lac**	**LiP**	**MnP**
*Bacillus thermoamylovorans 1141*	30–60	40	nd	1.7 ± 0.0	nd	nd	nd
*Geobacillus thermodenitrificans 3781*	20–60	50	nd	5.0 ± 0.3	nd	nd	nd
*Thermomyces lanuginosus 5718*	20–60	50	0.02 ± 0.01	37.1 ± 2.4	1.0 ± 0.2	0.3 ± 0.1	0.3 ± 0.1
*Aspergillus fumigatus 5731*	40–60	40	0.01 ± 0.00	54.6 ± 4.8	0.8 ± 0.3	0.2 ± 0.0	1.4 ± 0.4

*nd, not determined.*

In general, the Xyl production by bacteria was 10 times lower than that of the fungi, which is common when comparing both microbial groups (Alokika and Singh, 2019). Among the bacteria, *G. thermodenitrificans* 3781 produced more Xyl (5.0 ± 0.3 U/mL) than *B. thermoamylovorans* 1141 (1.7 ± 0.0 U/mL). *B. thermoamylovorans* is able to grow on xylan and the genome sequencing of a strain isolated from biogas plant–predicted genes involved in carbohydrate polymer degradation (Koeck et al., 2014), but no Xyl production has been reported to date; thus, this is the first description of such activity for this species. In contrast, members of *Geobacillus* are among the bacteria of choice for the levels of production of Xyls with stability at elevated temperatures (Chadha et al., 2019; Verma et al., 2019). For *G. thermodenitrificans*, Verma et al. (2020) reported Xyl production in the range of values obtained in the present work (5–10 U/mL) using xylan as carbon source. That value was further improved by optimization up to a maximum of 24 U/mL using complex substrates such as wheat straw as carbon source (Verma et al., 2020). In addition to *Geobacillus* species, *Caldicellulosiruptor*, *Thermopolyspora*, and several *Bacillus* species have been reported to be the more effective thermophilic bacteria for the production of thermostable Xyl (Bala and Singh, 2019; Chadha et al., 2019).

The two fungi produced all the enzymes tested, at reasonable levels of activity for Xyl in comparison to values reported in literature (Liu et al., 2013; de Gouvêa et al., 2018; Chadha et al., 2019) and very low for Cel and ligninases. This result was surprising, especially in the case of *T. lanuginosus*, known as a hyperproducer of thermostable Xyls that have no Cel and ligninase activity (Winger et al., 2014; Chadha et al., 2019). The production of Xyl by *T. lanuginosus* 5718 was low (37.1 ± 2.4 U/mL) in comparison to the reported values exhibited by some strains under optimized conditions. *T. lanuginosus* strains isolated from different environments vary in their Xyl production capacity, but in general, high Xyl activity is obtained, and optimization allows to dramatically increase Xyl activity. For example, *T. lanuginosus* isolated from decaying wood produced 132.5 U/mL using a combination of optimization approaches, whereas strain SSBP isolated from soil produced 3,575 U/mL, the highest reported activity of any known Xyl to date (Winger et al., 2014; Alokika and Singh, 2019; Chadha et al., 2019). The Cel (0.02 ± 0.01 U/mL), Lac (1.0 ± 0.2 U/mL), LiP (0.3 ± 0.1 U/mL), and MnP (0.3 ± 0.1 U/mL) activities exhibited by *T. lanuginosus* 5718, although being almost negligible, require further research as these enzymes have not been reported earlier for the species. Other thermophiles also exhibit very low values for Cels and ligninases as discussed below in the case of *A. fumigatus*. Gene expression assays are being conducted to validate the singular enzymatic activities encountered in *T. lanuginosus* 5718. *A. fumigatus* 5731 produced higher levels of Xyl (54.6 ± 4.8 U/mL) than *T. lanuginosus* 5718, but the production of Cel (0.01 ± 0.00 U/mL), Lac (0.8 ± 0.3 U/mL), LiP (0.2 ± 0.0 U/mL), and MnP (1.4 ± 0.4 U/mL) was similar, with very low values. For *A. fumigatus*, the production of all the enzymes has been reported to be very dependent on the carbon source used and the strain. The enzyme activities obtained are similar to those reported by Liu et al. (2013) that found a maximum Xyl activity of 15 U/mL after 2 days’ incubation of *A. fumigatus* Z5 on rice straw, whereas very low values of ligninases were detected. In contrast, de Gouvêa et al. (2018) obtained maximum activities of Cel (0.032 U/mL) and Xyl (10.82 U/mL) from steam-exploded bagasse. Although the analysis of the secretome revealed the presence of LIG-depolymerizing enzymes, no results on production were reported. The production of enzymes by *A. fumigatus* 5731 requires further optimization in order to reach values comparable to the production found by others. For example, *A. fumigatus* CWSF-7 produces 1.9 U/mL Cel (Mohapatra et al., 2018), whereas Lin et al. (2017) obtained high titers of Xyl (91.9 U/mL) and Cel (5.61 U/mL) activity after optimization when 1% barley straw was used as the carbon source.

## Conclusion

The present study provided insights into thermophilic culturome inhabiting composting material, which is involved in the degradation of lignocellulose. The study demonstrated that the whole fungal thermophilic population exhibits lignocellulose-degrading activity, of which only four species of Ascomycota are responsible. In the case of bacteria, 8–10% of the thermophilic population had this trait, although exclusively for HC degradation (xylan-degrading), which was due to a wider diversity dominated by representatives of the phylum Firmicutes. A common feature for bacteria and fungi was the capability to grow at a wide range of temperatures, having only a few representatives being strictly thermophilic. That allows them to survive and remain metabolically active over all stages of composting, playing a key role in the breakdown of HC during the entire process, with the degradation of CEL and LIG restricted to the activity of a few thermophilic fungi that appear at the end of the process. Likewise, the presence of phenotypes not previously described (either for thermal tolerance or for enzyme activities) within the species, besides the fact that many species evolve distinctly at the stages of composting, opens up many questions related to the mechanisms responsible for such adaptations to the changing composting ecosystem. The strains obtained in this work are a valuable resource to determine such mechanisms. Finally, the study selected some candidates representing a promising source of thermozymes with important biotechnological applications whose enzyme characteristics and production optimization remain to be determined.

## Data Availability Statement

The datasets presented in this study can be found in online repositories. This data can be found in López et al. (2021).

## Author Contributions

ML: formal analyses, investigation, supervision, and writing—review and editing. MJ: investigation and writing—original draft preparation. JL-G: methodology, formal analyses, and writing—original draft preparation. ME-G, MM-G, and AT: methodology and investigation. FS-E: methodology, visualization, and writing—original draft preparation. All authors contributed to the article and approved the submitted version.

## Conflict of Interest

The authors declare that the research was conducted in the absence of any commercial or financial relationships that could be construed as a potential conflict of interest.

## Publisher’s Note

All claims expressed in this article are solely those of the authors and do not necessarily represent those of their affiliated organizations, or those of the publisher, the editors and the reviewers. Any product that may be evaluated in this article, or claim that may be made by its manufacturer, is not guaranteed or endorsed by the publisher.

## Footnotes

1
http://www.ankom.com/procedures.aspx

2
www.bioinformatics.org/sms/revcomp.html

3
http://blast.ncbi.nlm.nih.gov/Blast.cgi
