# Supplementary material for: Characterization of Thermophilic Lignocellulolytic Microorganisms in Composting
Source: Front Microbiol. 2021 Aug 11;12:697480. doi: 10.3389/fmicb.2021.697480 (PMC8385673; doi:10.3389/fmicb.2021.697480)
Supplement: Supplementary file 1 [file Data_Sheet_1.pdf]

Supplementary Material

1 Supplementary Tables **Page**

1.	Table 1. Evolution of total and lignocellulolytic thermophilic bacteria (TB), actinobacteria (TA) and fungi (TF) counts (Log CFU/g d.w.), and lignocellulose fractions content (% d.w.) and degradation (%) at the stages of composting.	2
2.	Table 2. Taxonomic classification of thermophilic lignocellulolytic bacterial strains isolated during the composting process.	3
3.	Table 3. Taxonomic classification of thermophilic lignocellulolytic fungal strains isolated during the composting process.	4

Table 1. Evolution of total and lignocellulolytic thermophilic bacteria (TB), actinobacteria (TA) and fungi (TF) counts (Log CFU/g d.w.), and lignocellulose fractions content (% d.w.) and degradation (%) at the stages of composting. RM – Raw material; MES – Mesophilic; THER – Thermophilic; COOL – Cooling; FP – Final product.

**Parameter**	**RM**	**MES**	**THER**	**COOL**	**MAT**	**FP**
Total TB	8.12	9.60	9.28	8.80	7.96	7.79
Lignocellulolytic TB	7.81	8.87	8.69	7.76	7.36	7.00
Total TA	8.31	8.73	9.37	7.88	8.08	6.69
Lignocellulolytic TA	7.79	8.09	9.01	7.39	6.85	5.60
Total TF	2.00	3.95	3.28	2.00	3.99	0.00
Lignocellulolytic TF	2.00	3.65	3.28	2.00	3.99	0.00
Cellulose	28.22	23.21	25.62	24.02	15.1	7.2
Hemicellulose	4.43	7.41	4.63	4.91	4.0	6.5
Lignin	13.31	14.22	14.73	14.82	12.4	13.2
Holocellulose	32.61	30.62	30.21	28.93	19.0	13.7
Degradation Holocellulose	0.0	6.4	7.4	11.3	41.7	58.0
Degradation Lignin	0.00	0.0	0.0	0.0	6.7	6.8

d.w.: dry weight

Table 2. Taxonomic classification of thermophilic lignocellulolytic bacterial strains isolated during the composting process.

Classification	Number of strains
**Phylum: Actinobacteria**	
**Class: Actinobacteria**	
Family: Microbacteriaceae	
Microbacterium hydrocarbonoxydans	2
Microbacterium sediminis	2
**Phylum: Firmicutes**	
**Class: Bacilli**	
Family: Bacillaceae	
Aeribacillus pallidus	22
Bacillus aeolius	1
Bacillus aerius	6
Bacillus altitudinis	1
Bacillus amyloliquefaciens	1
Bacillus circulans	2
Bacillus coagulans	1
Bacillus licheniformis	49
Bacillus mojavensis	2
Bacillus niabensis	2
Bacillus pumilus	12
Bacillus safensis	4
Bacillus subtilis	8
Bacillus tequilensis	2
Bacillus thermoamylovorans	17
Bacillus thermolactis	1
Geobacillus stearothermophilus	2
Geobacillus thermodenitrificans	1
Lysinibacillus sphaericus	1
Terribacillus halophilus	1
Family: Paenibacillaceae	
Brevibacillus borstelensis	3
Paenibacillus ginsengihumi	10
Paenibacillus lactis	1
Family: Planococcaceae	
Ureibacillus thermosphaericus	2
**Phylum: Proteobacteria**	
**Class: Alphaproteobacteria**	
Family: Beijerinckiaceae	
Chelatococcus daeguensis	3
**Total number**	**159**

Table 3. Taxonomic classification of thermophilic lignocellulolytic fungal strains isolated during the composting process.

Classification	Number of strains
**Phylum: Ascomycota**	
**Class: Eurotiomycetes**	
Family: Aspergillaceae	
Aspergillus fumigatus	17
Family: Trichocomaceae	
Talaromyces thermophilus	1
Thermomyces lanuginosus	6
**Class: Sordariomycetes**	
Family: Plectosphaerellaceae	
Gibellulopsis nigrescens	3
**Total number**	**27**
